# A Comparative Overview of the Livestock-Environment Interactions in Asia and Sub-saharan Africa

**DOI:** 10.3389/fvets.2019.00037

**Published:** 2019-02-22

**Authors:** Joachim Otte, Ugo Pica-Ciamarra, Subhash Morzaria

**Affiliations:** Food and Agriculture Organization of the United Nations (FAO), Rome, Italy

**Keywords:** livestock, grassland degradation, pollution, water stress, biodiversity loss, greenhouse gases

## Abstract

Understanding the interactions between livestock and the environment in Asia and Sub-Saharan Africa is essential to sustainable livestock sector development. In this comparative overview, we review the available evidence on the extent of grassland degradation, land, and water pollution by nutrients and microorganisms, water stress, biodiversity loss, and greenhouse gas emissions and their relation to livestock production in Asia and Sub-Saharan Africa. We also draw on Asia's past livestock development trajectories and their impacts to provide guidance for future sustainable livestock development in Sub-Saharan Africa. Forward-looking policies and programs that anticipate long-term changes in the livestock sector and that assess trade-offs between policies and investments in multiple environmental domains in Sub-Saharan Africa are required to support sustainable development and guide policy decisions in the years ahead, from an environmental, social and public health perspective.

## Introduction

In the past two decades, and especially since the turn of the millennium, the African continent has been one of the fastest-growing regions of the world. Average annual gross domestic product (GDP) growth for the entire continent was over 4%, though with ups and downs and differences between countries (1). Economic growth prospects, both for the medium and long-term, are good (2–4). This anticipated development of the African continent will go hand in hand with transformative changes in its agriculture. The agricultural sector will transform not only to meet a spectacular increase in the demand for food, but also to satisfy the changing food preferences of an increasingly affluent and urbanized population. The sheer numbers are impressive: Africa's population is expected to reach 2.5 billion by 2050 vs. 1.2 billion today, with per capita consumption of food, as measured in kilocalories, more than doubling (5).

Within agriculture, the livestock sector is predicted to change dramatically. As GDP and consumer purchasing power grow, so will the demand for high-value products, including animal source foods such as meat, milk and eggs. In response, producers will invest in and expand livestock production and respective value chains, a phenomenon dubbed the “Livestock Revolution” (6). As in industrialized economies, the livestock sector, which currently accounts for about 1/3 of the value added of agriculture, is expected to become the largest contributor to agriculture.

The anticipated expansion of Africa's livestock sector and associated value chains may satisfy consumer demand but, if uncontrolled, could also have negative effects on public health, the environment and livelihoods, as experience elsewhere, for instance in Asia, has shown. In the last 30 years, meat consumption in South Asia, Southeast Asia and East Asia combined increased from about 36 to over 125 million tons and milk consumption from 60 to almost 220 million tons, that is by over 250% for both commodities. Parallel increases have occurred on the production side. For example, between 1985 and 2013 the poultry population passed from 3.5 to 12.4 billion and the off-take rate from 141 to 207% (7). This spectacular change in the livestock sector has been accompanied by a number of negative effects on society. Examples include smallholder farmers being squeezed out from commercial poultry and pig production (8); human health being threatened by outbreaks of zoonotic diseases, such as avian influenzas and animal food borne-diseases (9, 10); and by livestock-associated pollution of soil and water (11, 12).

**Figure 1:**
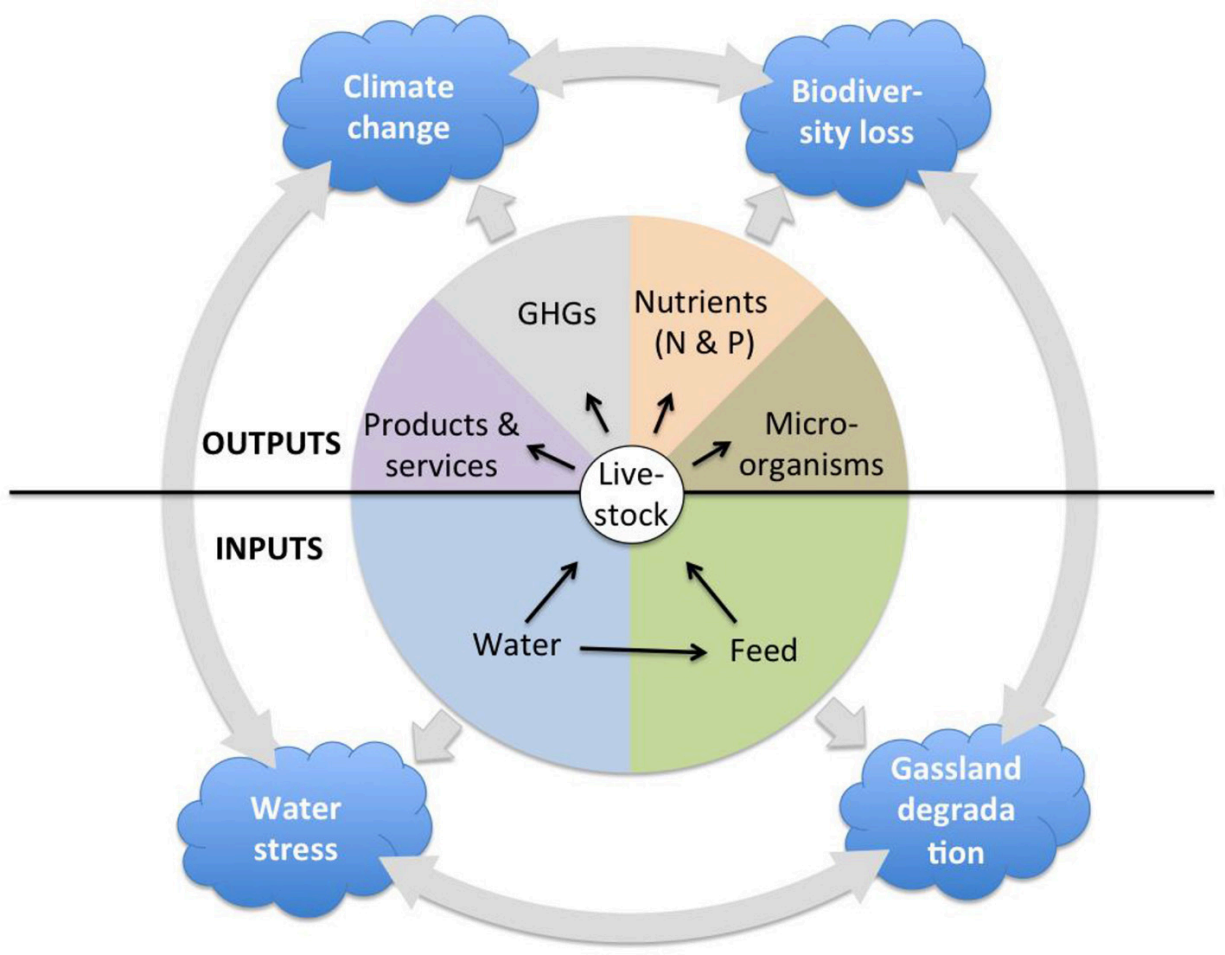
Schematic of livestock production and environmental impacts.

This paper aims to provide some guidance for sustainable livestock sector development in sub-Saharan Africa (SSA) by presenting a comparative overview of the relationship between livestock and the environment in SSA and Asia. There are a number of models used to assess the impact of livestock on a variety of environmental dimensions. For example, the Global Livestock Environmental Assessment Model (GLEAM) allows to investigate greenhouse gas emissions from livestock (13); Mekonnen and Hoekstra (14) use a linear equation to estimate the water footprint of livestock for all countries, which is calculated as the direct water footprint of drinking and service water and the indirect water footprint associated with feed production; Alkemade et al. (15) quantify the impact of livestock production on biodiversity by estimating changes in Mean Species Abundance of the original native species associated with the introduction of domesticated animals. There is no model, however, that comprehensively and jointly assesses the multitude of impacts of livestock on the environment. In this article, therefore, we provide a comparative overview of the extent of grassland transformation and degradation, land and water pollution by nutrients, and microorganisms, water withdrawal and stress, biodiversity loss, and greenhouse gas emissions, their relation to livestock production on the two continents, and the broad consequences of these environmental impacts (Figure 1).

Even though available literature and datasets only allow a broad exploration of the above livestock-environment interactions on the two continents, highlighting contrasts, and similarities generates useful information to guide the decision-making process.

## Grassland Transformation and Degradation

Grasslands, including rangelands and sown pastures, are among the largest ecosystems of the world and provide valuable ecosystems services. Grasslands sequester carbon, absorb methane, reduce emissions of nitrous oxide, and protect the soil from erosion. They thereby ameliorate local and regional climate and contribute to preserving the composition of the atmosphere. Overall, the value of ecosystem services provided by grasslands may be as large as, or larger than the sum of marketed products such as meat, milk, fibers, and hides from ruminant animals. Naturally, grasslands are mostly limited by water, but water availability permitting, they can be transformed into croplands. In sufficiently moist environments, most of the grasslands have in fact been converted into cropland while most of the grasslands in arid and semiarid environments remain as such and are largely, if not solely utilized to graze livestock.

Extensive areas of grassland are present both in Asia and SSA, comprising about 600 and 700 million hectares, respectively. Grasslands represent 80% of agricultural land in East Asia while they are far less prominent in South and Southeast Asia, where they only represent 25 and 13% of agricultural land, respectively (Tables 1, 2). In SSA, 77% of agricultural land is classified as grassland, ranging from 65% in Western Africa to 90% in Southern Africa.

**Table 1:** Total grassland area and grassland as proportion of total agricultural land in 1990 and 2010 by Asian sub-region and change 1990 to 2010.

	**Grassland area (million ha)**		**Proportion of total agricultural area (%)**		**Change 1990–2010 (%)**	
**Sub-region**	**1990**	**2010**	**1990**	**2010**	**Area**	**Prop**.
South Asia	94	78	28	25	−17	−13
East Asia	499	506	78	79	1	2
Southeast Asia	18	17	16	13	−5	−19

*Source: elaborated from FAOSTAT (Land)*.

**Table 2:** Total grassland area and grassland as proportion of total agricultural land in 1990 and 2010 by sub-Saharan Africa sub-region and change 1990–2010.

	**Grassland area (million ha)**		**Proportion of total agricultural area (%)**		**Change 1990–2010 (%)**	
**Sub-region**	**1990**	**2010**	**1990**	**2010**	**Area**	**Prop**.
Western Africa	172	184	71	65	7	−8
Eastern Africa	246	238	82	77	−3	−6
Central Africa	137	137	85	83	0	−2
Southern Africa	149	151	91	91	1	0

*Source: elaborated from FAOSTAT (Land)*.

In the last two decades, grassland areas have shrunk in Asia, while no major trends are recorded in the African continent. Specifically, between 1990 and 2010, grassland areas have decreased by 17 and 5% in South Asia and Southeast Asia, respectively, and remained stable (±1%) in East Asia (Table 1). They have decreased by 3% Eastern Africa, remained stable in Central and Southern Africa, and increased by 7% in Western Africa (Table 2). The latter increase has been linked to increasing rainfall in the Sahel over the last years (16, 17), while decreases of grassland areas in South Asia, Southeast Asia and Eastern Africa are due to conversion for other uses.

While reduction in grassland areas is more prominent in the Asian than the African continent, grasslands in Africa are experiencing higher degradation rates, with reduced soil fertility, plant diversity and productivity. Kwon et al. (18) estimated the global extent of degraded grasslands that were used for livestock grazing based on Normalized Difference Vegetation Index data from 2001 to 2010 corrected for annual rainfall. Their findings are that in East Asia around 77 million ha of grazing land are degraded while their figure for SSA is 340 million ha. Relating these estimates of the extent of degraded grazing areas to the amount of agricultural land classified as grassland by the Food and Agriculture Organization (FAO) yields degradation rates of 15 and 48% for East Asian and SSA grasslands, respectively. The latter value is in broad agreement with the estimate of Le et al. (19) of 40% of SSA's grassland being degraded. Within SSA, Eastern Africa experienced the most severe grazing biomass degradation with 65% of livestock grazing on degraded grasslands (20). In Eastern Africa, probably as an adaptation to rangeland degradation, pastoralists have changed herd structure from cattle to camels, sheep, and goats Ogutu et al. (21).

The causes of (grass) land degradation are numerous and complex. However, overgrazing by livestock prominently features among the cited causes. It reduces plant cover, and hence protection of the soil surface; it contributes to the degradation of riparian woods and forests, at the interface between land and water sources, with negative effects on water flows; it reduces the population sizes of wild herbivores and predators, which in turn affects biodiversity. The overall consequences are a reduction of provisioning and eco-systems services.

With respect to provisioning services, Kwon et al. (18) estimated that in SSA grassland degradation resulted in a loss of meat and milk production in 2007 of United States Dollars (USD) 812 million (a loss of USD 98/ton of grazing biomass dry matter reduction). In Kenya, the annual costs of rangeland degradation amounted to USD 80 million, with USD 24 million of losses experienced in warm arid and USD 16 million losses experienced in warm semi-arid agro-ecologies (22). For East Asia, production losses attributable to grassland degradation have been estimated as USD 145 million (a loss of USD 317/ton of grazing biomass dry matter reduction) (18).

For SSA, Nkonya et al. (20) estimate the total cost of land degradation on grazing biomass at about USD 1.11 billion, of which slightly more than half are attributed to reduced provisioning services while the remainder is attributed to reduced eco-systems services. For Niger, where grasslands represent 75% of non-desert land, the cost of the reduction of ecosystem services was estimated to be around USD 340 million in 2007, which represented around 5% of national GDP (23).

Grassland degradation can also affect people, who do not live from or in the proximity of the latter. For example, the increased frequency of sandstorms in China is clear sign of progressive desertification as direct consequence of degradation of the grasslands of northern China. Whereas, over the past century China was hit by almost 70 sandstorms, with an average frequency of one sandstorm every 3 years in the 1940s, the frequency had increased to one every 2 years by the 1960s. By the 1990s, the sandstorms in north China took place several times a year and this increased further to 12 in 2000 and 18 in 2001 (24). The frequency of sandstorms has since increased even further and, by carrying contaminants from the polluted soils of China's industrial north, severely affects air quality of major cities.

In SSA, grassland degradation warrants major attention given the large share of grasslands in SSA's agricultural area and the high rates of degradation (40% or more). Most of SSA's grasslands are managed by pastoralists, who are very knowledgeable about maintaining an ecological balance between pastures and livestock. However, pastoralism is particularly sensitive to population growth because the technical possibilities of changing the productivity of rangeland (changing the output-to-land ratio) are limited, especially when compared to yield increases obtainable by technical advances in crop production (25). Despite this limitation, Nkonya and Anderson (26) find that pastoral communities and other livestock keepers apply relatively sustainable land management practices if provided with good market access and secure land rights and if institutions are strengthened to regulate land use.

Given SSA's high population growth and associated growth in demand for animal source food, decision makers in the African continent should carefully assess current and future role of grasslands for society, including costs and benefits of transforming them into croplands. Grasslands' provision of marketed goods and services, which have a price tag, does not preclude the provision of other services, which may not be marketable. In fact, appropriate grassland management can enhance both aboveground biomass production (e.g., available to livestock) and belowground carbon sequestration (27). For SSA, overlooking grasslands' ecosystem services is likely to cause serious errors in resource allocation.

## Nutrient Overloading of Land and Water

Livestock dung is an important source of plant nutrients but can cause substantial pollution of ecosystems if managed improperly (28). Negative environmental consequences of excess nitrogen (N) and phosphorus (P) comprise reduced soil fertility, water eutrophication, contamination of groundwater with nitrates and bio-diversity loss. In 2007, the total amount of manure produced by livestock worldwide was estimated at 22.5 billion tons (29). Globally, the nutrient budget of agriculture as a whole is dominated by the rapidly growing livestock sector with its low nutrient recovery. Total nitrogen and phosphorus released into the environment through manure from livestock now exceed global nitrogen and phosphorus applied in fertilizer (30).

Historical changes and the possible future of nitrogen and phosphorus cycles in crop-livestock systems have been analyzed by 11. In many parts of Asia, nitrogen surplus currently exceeds 4,000 kg/km^2^/year while in SSA surpluses, where they exist, are mostly below 1,000 kg/km^2^/year (East African highlands have higher nitrogen surpluses). Overall, however, almost 80% of African countries are confronted with nitrogen scarcity or nitrogen stress problems (31) rather than nitrogen surpluses.

Table 3 presents estimates of the total amounts of nitrogen and phosphate (P_2_O_5_) excreted by livestock in the various sub-regions of Asia and SSA in 2010 and the resulting nutrient loads per ha of agricultural land. Per ha of agricultural land, nitrogen and phosphate from livestock are highest in South Asia, followed by Southeast Asia and East Asia, while in sub-regions of SSA nitrogen and phosphate loads are only between half and one third of those in Asia.

**Table 3:** Estimated amounts of N_total_ and P_2_O_5_ excreted by livestock by Asian and sub-Saharan Africa sub-region in 2010.

**Nutrient**	**Asia**			**Sub-Saharan Africa**			
	**South Asia**	**East Asia**	**Southeast Asia**	**Western Africa**	**Eastern Africa**	**Central Africa**	**Southern Africa**
**N**_**total**_							
1,000 tons	8,797	9,699	2,613	2,166	2,699	463	475
kg/ha ag. land	27.9	15.2	20.5	7.7	8.7	2.8	2.9
**P**_**2**_**O**_**5**_							
1,000 tons	3,382	5,086	1,258	895	1,023	183	186
kg/ha ag. land	10.7	8.0	9.9	3.2	3.3	1.1	1.1

*Source: Estimation based on livestock populations and annual excretions by animal type in Gerber et al. (32)*.

Gerber et al. (32) state, that “Phosphate (P_2_O_5_) overload is a concern in almost a fifth of cropland in South, East and Southeast Asia, mainly in eastern China, the Ganges basin and around urban centers such as Bangkok, Ho Chi Minh City, and Manila.” They estimate that livestock manure accounts for 39.4% of the agricultural phosphate supply (the remaining share being supplied by chemical fertilizers). Livestock manure is the main source of phosphate around urban centers and in areas specialized in livestock specialized production (southern and north-eastern China), while chemical fertilizers are dominant in crop (rice) intensive areas. In China, for example, direct manure discharge accounts for over two-thirds of nutrients in the northern rivers and for 20 to 95% of nutrients in the central and southern rivers (12). For SSA, MacDonald et al. (33) estimate that the median phosphorus balance is around 1 kg/ha/year (vs. ~10 kg/ha/year in Asia) and that most of the agricultural land falls either into the lowest deficit or lowest surplus quartile.

Nutrient overloads are a concern for the environment and public health. In the South China Sea, algal blooms caused by high phosphorus loads, including one in 1998 that killed more than 80% of the fish in 100 km^2^ along the coast of Hong Kong and southern China (34), are a recurring phenomenon. Due to high and increasing phosphorus loads of waters flowing into the South China sea, toxic algal blooms off the coast of China have expanded in geographic extent (from km^2^ to tens of km^2^), duration (days to months), and in harmful impacts (35).

Leaching of nitrate (NO_3_) from lagoons used for the storage of pig manure or from fields receiving an excess of livestock manure also affects ground / drinking water. In the Philippines and Thailand, for example, drinking water from 30% of all groundwater wells sampled showed nitrate levels above the World Health Organization safety limit of 50 mg/L of nitrate (36).

Given the livestock sector's predicted persistent growth, global nitrogen and phosphorus surpluses will continue to increase (+23% nitrogen and +54% phosphorus) despite improved nitrogen and phosphorus recovery in crop and livestock production (+35% nitrogen and +6% phosphorus recovery in crops and +35% nitrogen and phosphorus recovery in livestock). For Africa, Bouwman et al. (11) estimate that between 2000 and 2050 nitrogen and phosphorus surpluses per km^2^ will rise by 30 and 194%, respectively.

Pelletier and Tyedmers (37) estimate that in 2,000 global livestock production occupied 217% of the “safe space” for sustainable mobilization of reactive nitrogen and, based on FAO's global projections of demand for edible livestock products, predict that by 2050 the livestock sector may occupy 294% of the boundary for sustainable mobilization of reactive nitrogen. Manure accounts for approximately 40% of agricultural phosphorus use and the combination of fertilizer and manure is the primary driver of phosphorus surpluses in around 30% of the global cropland area (33). According to Rockström et al. (38) global phosphorus flows into the oceans have reached around 90% of the amount considered to be safe.

Asia's fulminant livestock development trajectory over the last decades has resulted in major nutrient overloads in soil and water. While driven by the necessity to provide an additional amount of food to a growing and increasingly affluent population, agriculture, and livestock intensification contributed to nutrient overloads, which are now a major concern from an environmental and public health perspective in the Asian continent. Although by 2,050 nutrient surpluses in Africa will still only be a fraction of those predicted for Asia, despite higher growth, African decision-makers should consider promoting livestock production models, which, while satisfying the growing demand for animal source foods, are ecologically sound and largely maintain (or even reinforce) the traditional links between livestock and the local resource base.

## Microbiological Pollution

Pathogenic microorganisms, which can cause various forms of gastrointestinal disease in humans and animals, can be considered as elements of soil and water pollution. Animal manure, in addition to nutrients, contains large numbers of microorganisms, which reside in the gastro-intestinal tract and are mostly part of the “normal” gut microbiome. Dairy cows for instance excrete 6 500 billion, feeder pigs 23 billion and layer chicken 1.8 billion coliform bacteria per day (39). Some of the excreted organisms may be pathogenic to other livestock species and humans and may be transported to water through surface runoff and erosion or by direct animal access to surface water. Streams and lakes used for drinking water supply and recreational purposes provide the greatest opportunity for transporting these pathogens to humans. Pathogens usually do not move through soil profiles and reach groundwater because of the filtering capabilities of soil. Exceptions to this may occur adjacent to poorly maintained well-casings (28). Untreated wastewater from slaughterhouses also contains large amounts of bacteria and microbial pollution around slaughter facilities is common [e.g., (40–42)].

In many parts of Asia, particularly South Asia, microbiological water quality has been found to be below World Health Organization (WHO) and national standards. In India for instance, nation-wide water quality monitoring indicated that 41% of surface water samples had fecal coliform counts exceeding the threshold of 500/100 mL and 14% of samples had fecal coliform counts of more than 5 000/100 mL (43, 44) examined the quality of well water in Karnataka, India, and found that 74 of 80 (92.5%) of the samples were contaminated with coliforms and 22 (27.5%) of the samples contained fecal indicator organisms. In Indonesia, Budisatria et al. (45) found high levels of well water contamination with fecal coliform bacteria in the vicinity of small ruminant housing. In Bangladesh, Ercumen et al. (46) compared the number of *E. coli* in water, soil and food in compounds with vs. without animals. *E. coli* was higher by 0.54 log_10_ in soil, 0.40 log_10_ in stored water and 0.61 log_10_ in food (*p* < 0.05) in compounds with animals. Over 50% of soil and 22% of stored water contained ruminant markers while the avian marker was detected in 33% of soil and 9% of water samples. In India, Schriewer et al. (47) detected animal fecal markers in 74% of ponds, 96% of households and 10% of groundwater drinking sources, indicating ubiquitous risks of exposure to zoonotic pathogens excreted with animal feces.

Microbiological assessments of water quality in various countries in SSA have also revealed significant levels of contamination. In Nigeria for instance, water from various sampling points on the Asa river all had TTC counts (Total Thermotolerant Coliform count, a surrogate for *E. coli*) >2 500/100 mL (48). Traoré et al. (49) found *Salmonella* in 15 and 20% of water samples from the reservoirs of Tanghin and Yamtenga, both of which serve Ouagadougou, Burkina Faso (*Salmonella* were commonly isolated from the fish (24%) caught from the reservoir of Tanghin and from the lettuce (50%) irrigated with water from Tanghin). In Ethiopia, Yasin et al. (50) report that 80% of water samples collected from unprotected water sources were positive for fecal coliforms with counts ranging between 0.7 and 267 colony forming units (CFU)/100 mL. Parker et al. (51) assessed microbiological water quality in 346 different water sources across the District of Amuria in Uganda. More than 90% of open wells and open water sources (*n* = 107) had TTC counts above the Ugandan national standard of 50/100 mL with median TTC counts above 1,000/100 mL. Substandard microbiological water quality has also been reported from Rwanda Sekomo Birame et al. (52), Kenya Kirianki et al. (53), and Zimbabwe Zvidzai et al.(54).

UNEP (55) has attempted to generate global statistics on water quality. Water samples from rivers in Africa have been found to have a median concentration of fecal coliform bacteria of 1,500 CFU/100 mL, i.e., water should be regarded as “severely polluted and unsuitable for any use,” while the median for Asia was 135 CFU/100 mL, i.e., the water is considered “suitable for agriculture as well as for bathing.” Consistently, regional annual mortality rates attributed to diarrhea from unsafe water, poor sanitation and hygiene in 2012 were highest for Africa, 43 deaths/100 000 population, followed by South Asia, with around 20 deaths/100,000 population, while in most East and Southeast Asian countries <5 deaths/100,000 population were attributed to water, poor sanitation and hygiene (56). Although these mortality rates primarily reflect the provision of access to safe drinking water, in regions where few people have access to safely managed drinking water services, e.g., 23% in SSA, they are also a reflection of the microbiological quality of surface and well water.

Identifying the source of pathogens in water (e.g., human waste vs. animal waste, livestock vs. wildlife excreta and waterfowl droppings) is challenging (57). However, given domestic animals such as poultry, cattle, sheep and pigs generate 85% of the world**'**s animal fecal waste and about 6 times the amount of waste produced by humans (29), and given the high microbiological loads of animal manure, particularly in young animals, of which some microorganisms represent human health hazards, livestock are highly likely to make a significant contribution to environmental pollution with potentially harmful microorganisms.

In particular, animal to human transmission via drinking water has been documented for several pathogens (e.g., *Cryptosporidium, Giardia, Salmonella*, and *Campylobacter*) and evidence exists of human health effects associated with exposure to recreational waters contaminated with animal and bird feces [e.g., (58–60)]. Prevailing standards of livestock manure management may thus not be appropriate for maintaining water safety.

Current indicator organisms, despite debates about their ability to represent the potential presence of pathogenic bacteria, consistently suggest that microbiological water pollution is significantly higher in SSA than in Asia and the foreseen expansion of livestock in the continent is likely to exacerbate this situation. African policy makers should consider ways to reduce prevailing levels of microbiological pollution from livestock and ensure that growth and transformation of the sector does not adversely affect future water quality, as sound management of freshwater ecosystems and access to safe water are essential for environmental sustainability and human health.

## Water Withdrawal and Stress

Livestock are a major user of water. Livestock production draws on water resources as drinking water, water to produce feed and water for cleaning and processing. The amount of drinking water used varies from 5 to 50 liters per Tropical Livestock Unit^1^ per day and depends on the species, dry matter intake, composition of the feed, water content of the feed, live weight of the animal, level of milk and meat production, physiological status of the animal and the climate in which the livestock is managed. However, the water required to produce daily feed for livestock is about 100 times the actual daily requirements for drinking water. Livestock typically require daily feed intake of dry matter amounting to about 3% of their weight and about 500 L (0.5 m^3^) of water is required to produce 1 kg dry matter (61). Thus, the impacts of livestock production on local/regional water balances largely depend on the predominant feeding systems, i.e., natural vegetation and/or crop residues vs. feed crops, and on feed production practices, i.e., rainfed vs. irrigated.

Water can be divided into blue (i.e., surface and groundwater), green (i.e., soil water), and gray (i.e., “consumed”) water. One of the main differences among various methods for assessing water use is whether and how they include green and gray water and the type of water used greatly determines the environmental impact of any activity. In addition to differences in water accounting methods, it is usually very difficult to disentangle water used for livestock production from water dedicated to other agriculture uses. Table 4, therefore, presents total agricultural water withdrawal by Asian and SSA sub-region without attempting to disaggregate between the livestock and other subsectors.

**Table 4:** Annual total (km^3^/year) and *per capita* agricultural water withdrawal (m^3^/year), its share in total water withdrawal (%) and its share in withdrawal of total renewable water resources (%) by Asian and sub-Saharan Africa sub-region in 2010.

**Agricultural water withdrawals**	**Asia**			**Sub-Saharan Africa**		
	**South Asia**	**East Asia**	**Southeast Asia**	**Western Africa**	**Eastern Africa**	**Central Africa**	**Southern Africa**
Total (km^3^)	913	469	328	48	17	1	17
p.c. (m^3^)	536	298	549	156	50	8	288
Agric. %	91	65	81	80	84	36	69
% renewable	46	21	7	5	7	<1	9

*Source: AQUASTAT and FAOSTAT (Human Populations)*.

In the Asian sub-regions, annual *per capita* agricultural water withdrawal ranges from around 300 to 550 m^3^, which is considerably higher than *per capita* agricultural water withdrawals in SSA. In Southern Africa, *per capita* withdrawal of 288 m^3^/year is similar to that of East Asia while withdrawals in Western, Eastern and Central Africa are much lower. In Asia, agriculture is responsible for two thirds to nine tenth of total water withdrawals and in East Asia water withdrawal is putting substantial pressure on water resources (>20% withdrawal of renewable water supply) while in South Asia the level of withdrawals has surpassed the threshold regarded as “critical” (>40% withdrawal of renewable water supply). In India, most irrigation water is used to grow food crops (~10% for cotton), whereas in China around 20% of groundwater is used for feed production (maize) (In the United States of America feed production, mainly fodder and maize, accounts for close to 50% of groundwater depletion). In SSA sub-regions, agriculture also accounts for the majority of water withdrawals (except in Central Africa), but water withdrawals are well within the limits regarded as “safe” (62).

Although currently SSA's water withdrawals are well within sustainable limits, increasing demand for livestock products and projected lower levels of rainfall due to climate change are predicted to lead to water scarcity in regions where it currently does not exist. The way the livestock sector will evolve, including in particular feeding practices, is going to have a major impact on water availability. Diminishing water supplies can translate into slower growth and reduced economic prospects. Under a business as usual scenario the World Bank (63) estimates that SSA, South and East Asia could see their growth rates decline by as much as 6% of GDP by 2050 as a result of water-related losses in agriculture, health, income, and property.

Sixty-two estimate that global freshwater use has reached 65% of the sustainability limit of 4,000 km^3^ per year with regions of high water overconsumption. Animal production is an important source of water consumption globally, accounting for almost one third of the blue and green water footprint (idem). As the contribution of agriculture to water scarcity is largely related to blue water use, blue water use has been suggested as the best criterion for estimating the influence of livestock on the risk of water scarcity (64). Alternatively, Atzori et al. (65) suggest the use of a “net water footprint index” (WFP_net_) for the quantification of water used for the production of animal products. The WFP_net_ offsets the water (blue and green) used for feed production by the water that would be used by the natural vegetation cover on the same land surface if crop production were abandoned. In situations where animals predominantly feed on natural vegetation blue water footprint and WFP_net_ are virtually identical.

Available evidence suggests that in East Asia livestock development has significantly contributed to increased water consumption. For China, for instance, Liu et al. (66) contend that the recent rise in meat consumption has pushed the country's annual *per capita* water requirement for food production up by a factor of 3.4 (from 255 m^3^ in 1961 to 860 m^3^). As China's freshwater resources amount to 2,220 m^3^ per person, just a quarter of the world average, the biggest threat to livelihoods and food security may be looming water shortages (67). In SSA, given the small percentage of irrigated land and the low use of feed crops, livestock are currently not important drivers of water withdrawal. However, as water is already scarce in large tracts of SSA, decision makers should be aware that future development trajectories of the livestock sector, particularly if associated with significantly increased production of feed crops, might greatly augment (local) water scarcity.

## Biodiversity Loss

Biodiversity, i.e., diversity within and among species and ecosystems, is one of the determinants of the supply of ecosystem services. Biodiversity loss has negative consequences for the functioning of ecosystems and its services. With regards to provisioning services, crop yields are increased by intraspecific genetic diversity, in plantations, production of wood is enhanced by tree species diversity, and in grasslands plant species diversity augments the production of fodder. With regards to regulating processes and services, increasing plant biodiversity increases resistance to invasive plant species, reduces the prevalence of plant pathogens, such as fungal and viral infections, increases aboveground carbon sequestration, and enhances soil organic matter (68).

The floral diversity of grasslands is surprisingly high, and the average areal richness of African savanna (≈1,750 species/10,000 km^2^) is not far below that of rain forest (≈2,020 species) [(69), cited by (70)]. Although commonly used for nomadic and transhumant pastoralism, African grasslands contain by far the widest variety of extant large and medium-sized herbivores (70). Traditional livestock keepers often maintain multi-species and multi-breed herds and flocks to increase their resilience and better cope with climatic and economic shocks (71).

While livestock are an element of biodiversity, depending on how they are managed, they may contribute to reducing biodiversity through a variety of mechanisms. Grassland degradation, nutrient overloads of land and water, high intensity of land use and land use change, particularly from primary vegetation to cropland or pasture, are leading to a significant reduction in biodiversity as measured by species richness and total abundance (72, 73). In 2014, the International Union for Conservation of Nature (IUCN) Red List registered 6 419 animal and 3 148 plant species in Africa as threatened with extinction (74). African vertebrate species are estimated to have declined by around 39% since 1970 (75). Species losses are more pronounced in Western and Central Africa than in Eastern or Southern Africa (76). UNEP-WCMC (77) does not provide similar quantitative information for Asia, but states, that “the region recorded the world's highest number of threatened species in 2014.”

There is evidence that Asia is experiencing higher biodiversity loss than Africa. Alkemande et al. (78) estimated relative mean species abundance, i.e., the existing abundance of original species relative to species abundance in undisturbed ecosystems, for different world regions for the year 2000. For SSA, they estimated a relative mean species abundance of 0.73 while for South and East Asia their estimate was 0.55, implying higher relative biodiversity loss in Asia than in SSA. However, their estimates indicate that both regions have experienced major losses in biodiversity over the past decades.

It is a challenge to quantify and value the cost for society due to biodiversity loss, for example because of the difficulties to attach a value to the life of any living organism. That said, there have been attempts to estimate the monetary value of the global welfare losses from the reduction of ecosystem services provided by land-based ecosystems. Between 2000 and 2010 these losses have been estimated to amount to Euro 545 billion, i.e., around Euro 50 billion losses per year, every year (79). Between 2010 and 2050, diminishing ecosystems services are projected to cost society another Euro 12 trillion (idem). With respect to ecosystems, reduced services from natural tropical forests and natural “grasslands” are predicted to cost society Euro 3.9 and 1.7 trillion respectively, i.e., almost half to the total global cost. From a regional perspective, estimates of land-based ecosystems service losses from 2000 to 2050 are Euro 2.4 trillion for Africa, Euro 0.8 trillion for China, and Euro 1.2 trillion for the remainder of South, East and Southeast Asia (idem).

Recorded extinctions of known species over the past 100 years indicate that current extinction rates are at least 100 times if not 1 000 times greater than rates characteristic of species in the fossil record (80). The ensuing dramatic reduction of biodiversity seriously threatens the functioning of ecosystems and their provision of services to human society. Costanza et al. (81) estimate the value of ecosystems services to have amounted to USD 125 trillion in 2011 (vs. global GDP of USD 73 trillion) and estimates of the cost of biodiversity loss are similar in magnitude to those of other global causes of environmental deterioration such as climate change or nutrient pollution.

As with most other aspects of environmental degradation, biodiversity loss results from the interplay of diverse component causes making quantitative attribution to a specific cause difficult. However, losses of wildlife in African grasslands are increasingly and primarily attributed to encroachment of agriculture and competition with livestock (21, 82, 83). Driven by rapid human population growth, the former leads to the reduction of grassland areas and their fragmentation while the latter accelerates grassland degradation (21). These effects are exacerbated by increased spatial and temporal variability of rainfall and increased frequency of droughts, and could be exacerbated by a growing livestock population, production intensification, and novel interactions between domesticate animals and wildlife.

As the long-term security of many ecosystem functions and services—especially in changing environments—is likely to depend upon local biodiversity, local population extinctions are more significant determinants of livelihood impacts than global extinction dynamics. Also, depending on location, the same factor can impact biodiversity through different mechanisms: in East Asia livestock production exerts substantial impact on biodiversity through its contribution to nutrient overloads, while in SSA livestock's impact on biodiversity is primarily mediated through grassland degradation. Furthermore, local pressures can lead to biodiversity impacts elsewhere, for instance, feed requirements for East and Southeast Asia's large livestock populations are a contributing factor to deforestation in South America.

Africa is still rich in biodiversity but, as in Asia, possible development trajectories, including livestock sector development, are likely to reduce plant and animal diversity. African decision-makers, therefore, should pay attention to the connection between livestock sector development and biodiversity when formulating policies and programs for livestock sector development. Gaining on the supply side while losing biodiversity, in fact, could ultimately be a zero, if not a negative sum game for society.

## Greenhouse gas Emissions

Greenhouse gases from human activities are the most significant driver of observed climate change since the mid-20th century (84). Combined, the three Asian sub-regions generated approximately 40% of total global greenhouse gas emissions (53.5 billion tons) in 2012 while SSA was responsible for <10% (85). Thus, SSA's contribution to climate change, which to a larger or lesser extent affects all world regions and countries, is relatively modest.

In 2010, agriculture, forestry and other land use generated approximately one quarter of total global greenhouse gas emissions^2^ (84). The contribution of agriculture, forestry and other land use to total greenhouse gas emissions varies by region and, in 2010, amounted to around 10% in the three Asian sub-regions vs. 33% in SSA (again with major sub-regional differences on both continents). In absolute terms, South, East and Southeast Asian agriculture emitted about 900, 760 and 450 million tons CO_2_-equivalent in 2010, respectively, while for Western, Eastern, Central and Southern Africa respective greenhouse gas emissions were 175, 300, 100, and 34 million tons CO_2_-equivalent (Tables 5, 6).

**Table 5:** Total agricultural greenhouse gas emissions and greenhouse gas emissions directly attributable to livestock production (gigagrams^a^ CO_2_ eq.) by source and Asian sub-region and growth of emissions (%) from 1990 to 2010.

**Sub-region**	**Source of greenhouse gas**	**1990**	**2010**	**Growth(%)**
South Asia	Agriculture	700,546	907,758	30
	Enteric ferm.	335,387	415,061	24
	Manure mgmt	32,489	41,360	27
	Manure on soils	17,902	24,426	36
	Manure on past.	82,323	109,047	32
	**Livestock total**	**468,101**	**589,894**	**26**
East Asia	Agriculture	615,610	763,980	24
	Enteric ferm.	155,617	222,595	43
	Manure mgmt	57,144	80,641	41
	Manure on soils	26,222	42,354	62
	Manure on past.	58,488	92,087	57
	**Livestock total**	**297,471**	**437,677**	**47**
Southeast Asia	Agriculture	331,347	445,598	34
	Enteric ferm.	59,115	72,219	22
	Manure mgmt	20,226	29,783	47
	Manure on soils	7,485	11,994	60
	Manure on past.	18,037	25,002	39
	**Livestock total**	**104,863**	**138,998**	**33**

a *1 gigagram = 1,000 tons Source: FAOSTAT (Emissions Agriculture)*.

**Table 6:** Total agricultural greenhouse gas emissions and greenhouse gas emissions directly attributable to livestock production (gigagrams^a^ CO_2_ eq.) by source and sub-Saharan Africa sub-region and growth of emissions (%) from 1990 to 2010.

**Sub-region**	**Source of greenhouse gas**	**1990**	**2010**	**Growth(%)**
Western Africa	Agriculture	109,033	176,066	61
	Enteric fermentation	39,226	71,518	82
	Manure management	2,938	5,563	89
	Manure on soils	1,317	2,595	97
	Manure on pasture	29,061	53,878	85
	**Livestock total**	**72,542**	**133,554**	**84**
Eastern Africa	Agriculture	213,807	300,492	41
	Enteric fermentation	86,486	128,250	48
	Manure management	4,349	6,851	58
	Manure on soils	1,812	3,209	77
	Manure on pasture	59,000	87,696	49
	**Livestock total**	**151,647**	**226,006**	**49**
Central Africa	Agriculture	88,866	99,996	13
	Enteric fermentation	13,843	19,508	41
	Manure management	1,201	1,880	57
	Manure on soils	568	913	61
	Manure on pasture	9,978	13,989	40
	**Livestock total**	**25,590**	**36,290**	**42**
Southern Africa	Agriculture	48,554	54,514	12
	Enteric fermentation	18,515	18,226	−2
	Manure management	1,030	1,107	7
	Manure on soils	433	507	17
	Manure on pasture	13,823	13,979	1
	**Livestock total**	**33,801**	**33,819**	**0**

a 1 gigagram = 1,000 tons.

*Source: FAOSTAT (Emissions Agriculture)*.

Within agriculture, in 2010, direct emissions from livestock production accounted for an estimated 65, 57, and 31% of emissions in South, East, and Southeast Asia, respectively (In Southeast Asia rice alone accounts for around 40% of agricultural emissions). In Western, Eastern, Central and Southern Africa respective values were 76, 75, 36, and 62%. Although livestock contribute higher shares to agricultural greenhouse gas emissions in SSA, in 2010, total greenhouse gas emissions from livestock in SSA amounted to <40% of those in Asia.

Ruminants, mainly through enteric fermentation and manure left on pasture, account for 90% or more of livestock's direct greenhouse gas emissions in South Asia and each of the four SSA sub-regions. In East and Southeast Asia the corresponding values are lower, but still around 70%. In all sub-regions, directly attributable greenhouse gas footprints of chicken are below 10% of all livestock-related greenhouse gas emissions.

On a *per capita* basis, livestock-related greenhouse gas emissions are highest in Eastern and Southern Africa, ~0.6 t CO_2_-equivalent/year, followed by Western and Central Africa with 0.4 and 0.3 t CO_2_-equivalent/year in 2010. In the three Asian sub-regions, *per capita* emissions related to livestock production are in the order of 0.2 t CO_2_-equivalent/year. Although in all regions, with the exception of Southern Africa, absolute amounts of livestock-related greenhouse gas emissions are growing in line with livestock sector growth, on a *per capita* basis they are declining, not only as in many sub-regions human population growth outpaces livestock sector growth, but also because livestock yields are improving and pig and poultry populations are growing faster than ruminant populations. Emissions per kg of animal protein by Asian and SSA sub-region are displayed in Table 7. Between 1990 and 2010, Asian sub-regions and Southern Africa have reduced their emissions per kg of product by 40% while in Western, Eastern and Central Africa emissions have declined by 10 to 18%.

**Table 7:** Total agricultural greenhouse gas emissions and greenhouse gas emissions directly attributable to livestock production (gigagrams^1^ CO_2_ eq.) by source in Asian and sub-Saharan Africa sub-region.

**Region & sub-region**	**kg CO**_****2****_**-eq/kg animal protein**		**Change**	
	**1990**	**2010**	**Abs**	**%**
**ASIA**				
South	129	75	−54	−42
East	52	31	−22	−41
Southeast	110	57	−53	−48
**SUB-SAHARAN AFRICA**				
Western	244	219	−25	−10
Eastern	281	230	−51	−18
Central	290	255	−35	−12
Southern	103	62	−41	−40

*Source: authors' calculations based on FAOSTAT for livestock production, protein content of animal products and greenhouse gas emissions from FAOSTAT*.

Climate change is manifest both in Asia and SSA. In Asia, surface air temperature has been rising while spatial as well as inter-seasonal and inter-annual variability in rainfall has increased. Rises in surface air temperature have been most marked in North Asia and higher in winter than in summer. Mean annual rainfall has decreased in Northeast and North China, Northeast India, Indonesia, some parts of Japan, the Philippines, and in coastal belts and arid plains of Pakistan. By contrast, Bangladesh, western China, China's Changjiang Valley, the southeastern coast of China, and the western coasts of the Philippines have experienced increases in mean annual rainfall (86).

For SSA, climate change projections point to a warming trend, particularly in the inland subtropics; frequent occurrence of extreme heat events; increasing aridity; and changes in rainfall, with a particularly pronounced decline in Southern Africa and an increase in Eastern Africa (87). Warming trends have already become evident across the continent, and it is likely that the continent's 2000 mean annual temperature change will exceed +2°C by 2100. Increasing temperatures will amplify existing water stress, putting additional pressure on agricultural systems, especially in semiarid areas, even if rainfall remains constant (88).

Climate variability will result in feed crop harvest quantities and quality becoming increasingly volatile (89). For soybeans, for instance, the predicted impact of climate change on yields in 2050 ranged from −43 to +10%, depending on climate model and adaptation scenario (90). Without adaptation, yields were generally predicted to decline, however with considerable spatial variation. With respect to maize, a systematic literature review by Knox et al. (91) found that for Africa and South Asia predicted yields declined by 5 and 16% respectively, from 2030 to 2100. Feed scarcity and associated sharp increases in feed costs may therefore force animal production to rely more on crop residues and by-products of food destined for human consumption and / or slow down the growth of pig and poultry production.

Although SSA's *per capita* emissions are only around 2.4 tons CO_2_-equivalent/year vs. the global average of 6.2 (European Union around 10, United States of America around 21), 7 of the 10 countries most vulnerable to climate change are located in SSA (92). One of the unifying characteristics of these countries is that they depend heavily on agriculture, with 65% of their combined working population employed in the sector and close to 30% of their overall economic output derived from agricultural revenues.

Greenhouse gas emissions per kg of animal protein are five times as high in Western, Eastern and Central Africa than in East and Southeast Asia, and three times as high as those in South Asia. These high emissions per kg of animal protein in Western, Eastern and Central Africa are the result of the large share of extensive and semi-intensive ruminant production in the respective livestock sectors. It should be noted that the above are estimates of gross greenhouse gas emissions and do not take into account CO_2_-equivalent sequestered by the vegetation on which animals are raised (above as well as belowground). For instance, Barretto de Figueiredo et al. (93) estimated that the carbon footprint of beef cattle on managed pastures was reduced from 9.4 to 7.6 kg CO_2_-equivalent per kg live weight if soil carbon sequestration was included. Integrated crop-cattle-forest systems stored 28.1 kg CO_2_-equivalent per kg live weight thereby acting as net carbon sink (idem).

The global potential to reduce greenhouse gas emissions from the livestock sector has been estimated as 2.4 billion tons CO_2_-equivalent per year, i.e., by about one third. The vast majority of this potential is associated with ruminant species, which are responsible for over 90% of all direct greenhouse gas emissions from livestock. In spite of this potential, few studies have assessed the costs and benefits of different greenhouse gas mitigation practices. Those that have, suggest that between 0.2 and 0.6 billion tons CO_2_-equivalent of this potential (<10% of livestock emissions and <2% of total emissions) can be realized at a price of USD 50 per ton CO_2_-equivalent per year, the amount that polluters must pay for their emissions, or be paid to polluters to reduce their emissions (94). This is significantly lower than the total abatement potential, which suggests much of the theoretical potential is not attainable in a cost-effective manner.

Even though a significant reduction of SSA's livestock-derived greenhouse gases would have a minimal impact on global greenhouse gas emissions (as they currently represent <1% of global total emissions), they warrant some consideration by African decision-makers because of their importance in international climate change negotiations. Given the strong demand growth for animal source food, SSA's potential for absolute reduction in greenhouse gas emissions from livestock may be limited, but there is ample scope to reduce greenhouse gas emissions per kg of animal protein through improved grassland management and intensification to reach levels similar to those in South Asia.

## Conclusion

Africa has been one of the fastest-growing economic regions of the world in the past decades and prospects for the future are good because of exponential population growth; gains in real per capita income; rapid urbanization; technology adoption; political vision and commitment. The continent is thus expected to dramatically change in the coming decades, including its agriculture and, within agriculture, its livestock sector. As GDP and consumer purchasing power grow, so will the demand for animal source foods such as meat and milk. The anticipated expansion of livestock production and associated value chains is expected to largely satisfy consumers' demand, but could also have negative effects on public health, the environment and livelihoods. Generating evidence on the trade-offs of livestock sector development, therefore, is essential for decision-makers to take effective policy decisions.

As of today, the impact of livestock on the environment is by far less pervasive in SSA than in Asia, but the anticipated expansion of livestock production and associated value chains, if uncontrolled, is very likely to have negative effects on the environment from soil through water to air and biodiversity. SSA decision-makers should be aware and draw lessons from the past trends of livestock growth in Asia to design and implement policies that effectively manage the trade-offs associated with livestock sector transformation, including environment, livelihoods and public health dimensions. Researchers should facilitate informed decisions not only by refining existing models that generate evidence on the impact of livestock on selected environmental domains, such as soil, water, air and biodiversity, but also by developing models that assist in simultaneously and systematically assess the trade-offs of livestock sector policies and investments on all major environmental dimensions, which would allow the efficient allocation of scarce resources for a sustainable development of livestock in the long-term.

## Author Contributions

All authors listed have made a substantial, direct and intellectual contribution to the work, and approved it for publication.

### Conflict of Interest Statement

The authors declare that the research was conducted in the absence of any commercial or financial relationships that could be construed as a potential conflict of interest.

## Footnotes

^1^Tropical Livestock Units are livestock numbers converted to a common unit. Commonly used conversion factors are: cattle = 0.7, sheep = 0.1, goats = 0.1, pigs = 0.2, chicken = 0.01.

^2^This estimate does not include the CO_2_ that ecosystems remove from the atmosphere by sequestering carbon in biomass, dead organic matter, and soils, which offset approximately 20% of emissions from this sector (IPPC, 2014).
